# Autologous Dendritic Cell Therapy in Mesothelioma Patients Enhances Frequencies of Peripheral CD4 T Cells Expressing HLA-DR, PD-1, or ICOS

**DOI:** 10.3389/fimmu.2018.02034

**Published:** 2018-09-07

**Authors:** Pauline L. de Goeje, Yarne Klaver, Margaretha E. H. Kaijen-Lambers, Anton W. Langerak, Heleen Vroman, André Kunert, Cor H. J. Lamers, Joachim G. J. V. Aerts, Reno Debets, Rudi W. Hendriks

**Affiliations:** ^1^Department of Pulmonary Medicine, Erasmus MC, Rotterdam, Netherlands; ^2^Erasmus MC Cancer Institute, Erasmus MC, Rotterdam, Netherlands; ^3^Department of Medical Oncology, Laboratory of Tumor Immunology, Erasmus MC, Rotterdam, Netherlands; ^4^Department of Immunology, Laboratory Medical Immunology, Erasmus MC, Rotterdam, Netherlands

**Keywords:** dendritic cell vaccination, mesothelioma, immune monitoring, T lymphocytes, immunotherapy of cancer, inducible T-cell co-stimulator protein, programmed cell death 1 receptor

## Abstract

**Introduction:** Malignant pleural mesothelioma (MPM) is a malignancy with a very poor prognosis for which new treatment options are urgently needed. We have previously shown that dendritic cell (DC) immunotherapy provides a clinically feasible treatment option. In the current study, we set out to assess the immunological changes induced by DC immunotherapy in peripheral blood of MPM patients.

**Methods:** Peripheral blood was collected from nine patients enrolled in a phase I dose escalation study, before and after treatment with DCs that were pulsed with an allogeneic tumor lysate preparation consisting of a mixture of five cultured mesothelioma cell lines. We used immune profiling by multiplex flow cytometry to characterize different populations of immune cells. In particular, we determined frequencies of T cell subsets that showed single and combinatorial expression of multiple markers that signify T cell activation, maturation and inhibition. Therapy-induced T cell reactivity was assessed in peptide/MHC multimer stainings using mesothelin as a prototypic target antigen with confirmed expression in the clinical tumor lysate preparation. T cell receptor (TCR) diversity was evaluated by *TCRB* gene PCR assays.

**Results:** We observed an increase in the numbers of B cells, CD4 and CD8 T cells, but not NK cells at 6 weeks post-treatment. The increases in B and T lymphocytes were not accompanied by major changes in T cell reactivity toward mesothelin nor in *TCRB* diversity. Notably, we did observe enhanced proportions of CD4 T cells expressing HLA-DR, PD-1 (at 2 weeks after onset of treatment) and ICOS (6 weeks) and a CD8 T cell population expressing LAG3 (2 weeks).

**Discussion:** DC immunotherapy using allogeneic tumor lysate resulted in enhanced frequencies of B cells and T cells in blood. We did not detect a skewed antigen-reactivity of peripheral CD8 T cells. Interestingly, frequencies of CD4 T cells expressing activation markers and PD-1 were increased. These findings indicate a systemic activation of the adaptive immune response and may guide future immune monitoring studies of DC therapies.

## Introduction

Malignant pleural mesothelioma (MPM) is a solid tumor of the pleural lining that is strongly related to the exposure to asbestos (1). Overall survival is poor with a median survival of less than a year, and conventional therapies like chemotherapy and radiotherapy being able to improve survival only by a few months (2). Immunotherapy has evolved as an important new treatment modality in various kinds of cancer, with checkpoint inhibitors currently being FDA approved as first line therapy for several cancer types. Initial results of clinical studies for MPM with checkpoint inhibitors as second line treatment showed response rates of 9–25% (3).

MPM is characterized by a strong immunosuppressive component, with relatively low numbers of T cells infiltrating the tumor (1, 4). These low numbers of tumor-infiltrating T cells have prognostic value in MPM (5) and might explain the relatively low response rates to checkpoint inhibitors (1).

Furthermore, in MPM patients dendritic cells (DCs) have been shown to be reduced in numbers and in antigen-processing function compared to healthy controls, which negatively affected survival outcomes (6). The reduced functionality of DCs is thought to relate to low intra-tumoral T cell numbers. Along these lines, DC vaccination represents a promising therapeutic strategy.

Previously, we have developed a cellular therapy for MPM, consisting of autologous DCs pulsed with autologous tumor lysate with the intention to cover a broad range of tumor antigens (7). This vaccination strategy was shown to be safe with promising clinical outcomes (7, 8). However, the availability and quality of tumor material that could be obtained, limited the feasibility of the treatment with DCs loaded with autologous tumor lysate. To overcome this limitation, DC vaccination using allogeneic tumor lysate was developed and tested for safety and feasibility in a phase I clinical trial (9). Allogeneic tumor lysate derived from five *in vitro* cultured clinical-grade human mesothelioma cell lines was used to pulse autologous DCs and the resulting DC vaccine was administered to patients i.d. and i.v. once every 2 weeks for three cycles, with a booster vaccination at 3 and 6 months after the start of treatment. The study was set up as a dose escalation study with three cohorts of three patients, and each cohort received 10 million, 25 million or 50 million DCs per vaccination, respectively. By circumventing the immunosuppressive tumor immune environment and providing enhanced tumor antigen presentation with DC vaccination, impressive objective responses could be obtained, as exemplified by a tumor reduction of ~70% at 6 weeks post-treatment in one of the patients in this phase-I trial (9).

In the current study we aimed to characterize the immunological changes induced by DC immunotherapy in these nine MPM patients. For a better understanding of the immunological changes induced by DC immunotherapy we monitored peripheral blood, which is the preferred compartment for sequential sampling. We used extensive multiplex flow cytometry with a focus on T cell activation and inhibitory markers and characterized T cell specificity using peptide-MHC multimers to obtain a detailed immune profile and immune dynamics following DC immunotherapy.

## Methods

### Patients

The nine patients in this study participated in a first-in-human clinical trial as described by Aerts et al. (9). In short, all patients had pathologically-proven MPM and were included in the study at least 6 weeks after their last chemotherapy treatment, or were treatment-naive if they had refused chemotherapy treatment. After inclusion in the study, patients received leukapheresis, which was used as a source of autologous DCs.

The DCs were prepared as described (9) and pulsed with a lysate, consisting of a mixture of five *in vitro* cultured mesothelioma cell lines. Patients received a total of three vaccinations every 2 weeks and blood samples were obtained at baseline and at week 2, 4, 6, and 8 following initial vaccination. Booster vaccinations were administered at 3 and 6 months (9). One third of the dose was administered intradermally (i.d.), and two thirds of the dose intravenously (i.v.). As this was a dose escalation study, patients 1–3 received 10 million DCs per vaccination, patients 4–6 received 25 million DCs per vaccination and patients 7–9 received 50 million DCs per vaccination. Patients 7 and 9 did not receive their second booster vaccination due to shortage of patient material. All other patients completed the full treatment scheme (Table S1). For flow cytometry (FCM) analysis, cohort 1 was not included since the collected peripheral blood samples of patients in cohort 1 were immediately processed and stored. For cohort 2 and 3 the protocol was amended to enable absolute immune cell quantification.

### Collection and processing of peripheral blood samples

Ethylene diamine tetra acetic acid (EDTA) anticoagulated peripheral blood was drawn from patients at baseline prior to the first vaccination (week 0), at 2 weeks after the first vaccination, i.e., prior to the second vaccination (week 2) and 2 weeks after the third vaccination at week 6 and analyzed within 6 h by multiplex FCM. One ml of whole blood was used for multiplex FCM and from the remaining blood, peripheral blood mononuclear cells (PBMCs) were isolated by standard Ficoll density gradient centrifugation and were stored at −80°C for further analyses.

### Multiplex flow cytometric assessment of numbers and phenotype of immune cells

To enumerate immune cell populations, whole blood (100 μl) was stained with the “absolute numbers” panel (Table S2) and incubated for 15 min at room temperature. Subsequently, 2 ml of lysis buffer (NH_4_Cl: 8.26 mg/ml, KHCO_3_: 1 mg/ml and EDTA: 37 μg/ml) was added to the blood and incubated for 15 min at room temperature. Subsequently 100 μl of Flow-Count Fluorospheres (Beckman Coulter Inc.) was added and samples were measured on a BD LSRFortessa™ flow cytometer. A minimum of 10,000 CD45^+^ cells were measured to enable clear distinction of defined immune cell populations. Subsequently, data was analyzed with FlowJo version X (FlowJo, LCC) using the gating strategy as exemplified in Figure S1. Values were expressed as cells per microliter. To determine the phenotype of T cells, the T cell maturation, activation, co-inhibitory and co-stimulatory receptors were analyzed on whole blood with different FCM panels (Table S2). 100 μl of whole blood was stained with each of the panels and incubated for 15 min at room temperature. Subsequently, 2 ml of lysis buffer was added to the blood and after an incubation of 15 min at room temperature, the cell suspensions were centrifuged at 450 g for 5 min, washed and resuspended in buffered 0.1% paraformaldehyde (PFA). A minimum of 30.000 CD3^+^ cells were measured on the LSRFortessa™ flow cytometer to obtain clearly detectable immune populations. The FCM data were analyzed with FlowJo version X.

### Determination of mesothelin-specific T cells

PBMCs were thawed and subsequently T cells were rapidly expanded with a feeder system as described elsewhere (10). After this rapid expansion protocol, T cells were co-cultured with artificial antigen presenting cells (aAPC) (11) loaded with mesothelin peptide A: SLLFLLFSL (mesothelin_20−28_), or mesothelin peptide B: VLPLTVAEV (mesothelin_531−539_; Immudex, Copenhagen, Denmark). The aAPC cell line is based on K562 cells, retrovirally transduced with CD80, CD83, and HLA-A2 for optimal antigen presentation and co-stimulation, and enables enrichment of antigen-reactive T cells with protocols optimized in our laboratory (12). 2.5 × 10^6^ aAPC cells/ml were incubated at room temperature for 5 h with 10 μg/ml peptide and subsequently irradiated (120 Gy). Subsequently, these peptide-loaded aAPC cells were co-cultured with T cells (ratio 1:20) in T cell medium (RPMI Hepes [Lonza] supplemented with 10% human serum [Sanquin, Amsterdam, The Netherlands], 1% L-glutamine and 1% penicillin/streptomycin), 180 IU IL-2 per ml (Chiron, Amsterdam, The Netherlands), and 5 ng IL-15 per ml (Le-Perray-en-Yvelines, France). Mesothelin-driven T cell expansion was continued for four cycles, after which T cells were examined for their binding ability to corresponding mesothelin peptide-HLA-A2 complex multimers. In this study, we performed a maximum of four peptide-specific expansion cycles.

Dextramer A: PE-conjugated HLA-A2 dextramer with peptide SLLFLLFSL (mesothelin_20−28_) and dextramer B: APC-conjugated HLA-A2 dextramer with peptide VLPLTVAEV (mesothelin_531−539_) were both ordered from Immudex (Copenhagen, Denmark). Dextramer staining was performed according to manufacturer's protocol. Anti-CD3-BV711 (clone UCHT1) and anti-CD8-FITC (clone SK1) mAbs (both from BD Biosciences) were used together with dextramers for extracellular staining. 4′,6-diamidino-2-phenylindole (DAPI) was used as viability dye. Samples were measured on an LSR-II flow cytometer (BD). Fluorescence-minus-one (FMO) controls were used to enable gating and determine dextramer-positive populations.

### GeneScan T cell clonality analysis

Cell pellets from PBMC samples were frozen and stored until further use. Genomic DNA was isolated using the AllPrep DNA/RNA Mini kit (QIAGEN, Hilden, Germany) according to manufacturer's instructions. T-cell receptor (TCR) β gene repertoire was measured using commercially available multiplex TCR Vβ-Jβ PCR assays (Invivoscribe, San Diego, CA, USA) as developed and approved by the BIOMED-2/EuroClonality consortium (13). GeneScan fragment analysis was done on an ABI 3130 xl instrument (ThermoFisher Scientific) and data were analyzed using PeakScanner software. Data interpretation was based on GeneScan patterns of duplicate PCR results, largely following the EuroClonality guideline (14).

### DC-PBMC co-cultures

Mature lysate-pulsed DC (as were used for the DC immunotherapy) were co-cultured with PBMC samples of the same patient. For each patient PBMC samples from week 0, week 2, and week 7 were co-cultured with DCs in a 96-wells plate in a 1:10 ratio, in RPMI supplemented with 10% normal human serum (duplicate cultures). After 24 h, the supernatant was collected for enzyme-linked immunosorbent assay (ELISA) to determine IFNγ (Invitrogen) and the cells were harvested for flow cytometry. For flow cytometric analysis, the cells were stained with the following antibodies: anti-CD3-APC-eF780 and anti-CD8-AlexaFLuor700 (eBiosciences), anti-CD4-BV785, anti-CD56-PE-Cy7 and anti-CD69-FITC (BD Biosciences) and anti-CD137-PE (Biolegend).

### Statistical analyses

Statistical analyses and graphs were made with Graphpad Prism v5.0. For comparison of changes from baseline measurements, Wilcoxon signed-ranks test was used to test for significance between baseline measurements and other time points. The heatmap was made in R for windows version 3.4.1 using the package in “gplots” (https://cran.r-project.org/web/packages/gplots/). Clustering was performed with a complete agglomeration method, and distance matrix of all variables was computed with Euclidean distance.

## Results

### Numbers of B, CD4 and CD8T lymphocytes in peripheral blood increase upon vaccination with DCs

**Figure 1:**
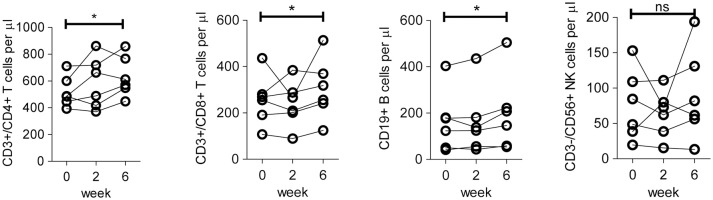
Absolute number of lymphocyte subsets in peripheral blood of patients before and after DC immunotherapy. Quantification of absolute numbers of CD4 T cells (CD45^+^/CD3^+^/CD4^+^), CD8 T cells (CD45^+^/CD3^+^/CD8^+^), B cells (CD45^+^/CD3^−^/CD19^+^), and NK cells (CD45^+^/CD3^−^/CD56^+^) in peripheral blood of patients in cohort 2 and 3 on baseline prior to the first vaccination (week 0), 2 weeks after the first vaccination, i.e., prior to the second vaccination (week 2) and 2 weeks after the third vaccination (week 6). Differences between week 0 and week 6 with respect to paired continuous parameters were determined using the exact Wilcoxon signed rank test. ^*^p < 0.05; ns, not significant.

We first assessed the absolute numbers of specific lymphocyte subsets (CD3^+^CD4^+^, CD3^+^CD8^+^, CD3^−^CD19^+^ and CD3^−^CD56^+^) from the six patients treated in the second and third cohort (dosage 25 and 50 million DC per vaccination), using the gating strategy as shown in Figure S1. Patients showed a significant increase at week 6 (after three vaccinations) compared to baseline in (median) numbers of CD4^+^ T cells (increase from 522 to 634 cells/μl), CD8^+^ T cells (from 256 to 304 cells/μl) and B cells (from 162 to 199 cells/μl), but not Natural Killer (NK) cells (Figure 1). Together, these results are indicative for a potentiation of adaptive immunity in peripheral blood after DC immunotherapy.

### Lysate-specific and mesothelin-specific T cells are detectable in peripheral blood of mesothelioma patients

Next, we investigated whether the increased numbers of T cells due to DC vaccination harbored vaccine-specific CD8 T cells. For three patients, lysate-pulsed DCs were available after completion of the treatment schedule. Co-cultures of PBMC before and after treatment with these lysate-pulsed DC showed an increase in CD69-positive CD4^+^ and CD8^+^ T cells, CD137-positive CD4^+^ and CD8^+^ T cells, and IFNγ secretion after therapy, supporting the induction of a vaccine-specific response (Figure S2).

**Figure 2:**
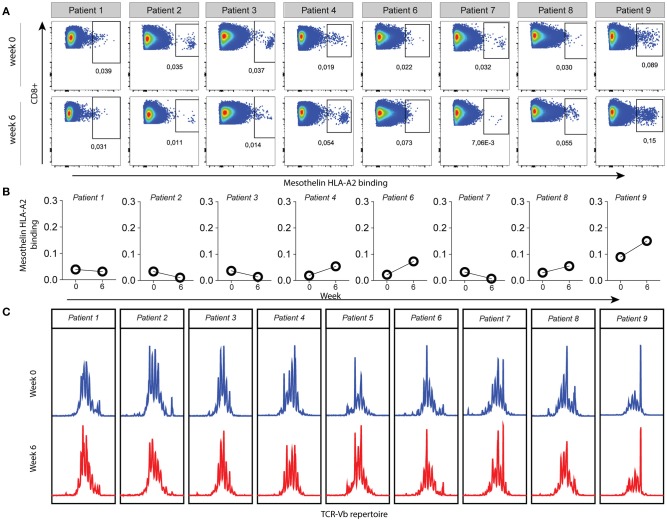
Mesothelin-specific CD8 T cells in HLA-A2 positive patients are measurable in pre- and post-vaccination samples. **(A)** Peripheral blood samples were collected at baseline (week 0) and 2 weeks after the third vaccination (week 6). CD8 T cells were propagated in four culture cycles with mesothelin-peptide-B loaded aAPC, after which mesothelin-peptide B/HLA-A2 dextramers were used to detect mesothelin-specific CD8 T cells. Gating was based on the negative controls, FMO staining and the non-CD8 T cell population. All HLA-A2 positive patients are shown; patient 5 was excluded (haplotype HLA-A3/HLA-A68). **(B)** Values of gated dextramer-binding CD8 T cells as shown in **(A)** presented as proportions of total CD8 T cells. **(C)** Relative frequencies of complementarity determining region-3 (CDR3) lengths in pre- and post-therapy total T cells from PBMC samples of all patients. The Y-axis represents relative frequency as assessed by fluorescence intensity, with the various CDR3 lengths on the X-axis. A polyclonal repertoire would follow a normal distribution.

To further identify vaccine-specific CD8 T cells in all patients we selected mesothelin as a prototypic antigen based on the following lines of evidence. Firstly, RNA sequencing and western blot data obtained from the mesothelioma cell lines used to generate the lysate preparation validated mesothelin mRNA and protein expression. Secondly, immune histochemistry confirmed the expression of mesothelin in eight out of eight available patient biopsies. Lastly, mesothelin peptide/HLA-A2 complexes with reported immune reactivity (15, 16) (peptide A: SLLFLLFSL and peptide B: VLPLTVAEV) were bound by CD8 T cells derived from patient skin biopsies after challenge with DC vaccine (9), as summarized in Table S2. To determine whether DC therapy would induce changes in the frequency of mesothelin-specific CD8 T cells, we measured the binding of two mesothelin-peptide/HLA-A2 multimers by CD8 T cells in pre- vs. post-vaccination peripheral blood samples. To this end, T cell fractions were first propagated using four T cell culture cycles in the presence of mesothelin peptides (A and B). Figures 2A,B show flow cytometry plots for one of the two mesothelin epitopes (peptide B) in propagated CD8 T cell fractions from HLA-A2-positive patients (8 out of 9 patients). These analyses show that the frequency of mesothelin-specific CD8 T cells is variable among patients, and already pre-exists in 5 out of 8 HLA-A2 positive patients and does not change significantly upon treatment. Similar data were obtained for a second mesothelin epitope (peptide A; Figure S3) and no correlation was observed between the frequencies of CD8 T cells specific for the two epitopes analyzed (data not shown).

In addition, we studied TCRB diversity of total T cells as a global measure of T cell reactivity via GeneScan TCRB PCR (Figure 2C). While some patients showed dominant peaks (e.g., patient 9), the GeneScan patterns were generally similar in pre-treatment and post-treatment PBMC fractions from the MPM patients, indicating that DC therapy did not induce clonal T cell expansion or selection. Collectively, these findings show that DC vaccination-specific T cells are detectable, but that this is not accompanied by an overall enhancement of frequencies in peripheral blood, nor obvious shifts in T cell TCRB repertoire.

### Increased frequencies of PD-1, HLA-DR and icos positive CD4^+^ T cells after DC vaccination

**Figure 3:**
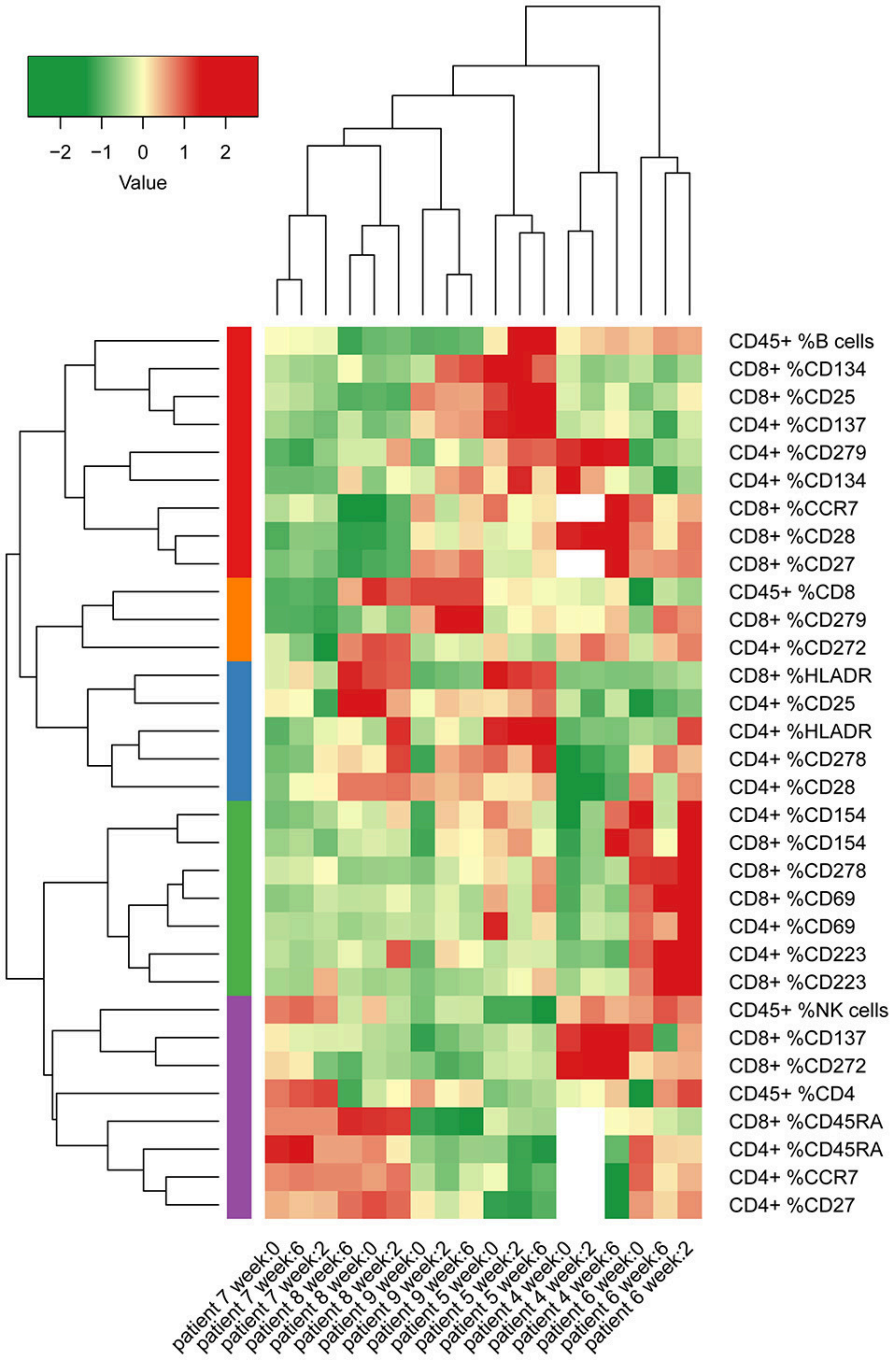
Heatmap of percentages of several different lymphocyte subsets and T cell surface markers in peripheral blood from patients in cohort 2 and 3 (MCV004–MCV009). Columns represent different patient samples at week 0, week 2, and week 6 after start of treatment. Rows represent the proportions of CD4 T cell, CD8 T cell and CD45 lymphocyte populations that express defined markers. Two of the co-inhibitory molecules in our panel, CTLA-4 and TIM 3 were expressed at too low frequencies to yield reliable values. Therefore, we removed these markers from further analysis. Percentages were normalized according to mean values of all measurements and were clustered by non-hierarchical clustering.

We then evaluated changes in frequencies of CD4 or CD8 T cells expressing surface activation, maturation or co-signaling markers and compiled a heatmap of all variables measured by FCM at pre- and post-therapy time points (Figure 3). Hierarchical clustering of samples showed clustering of the different time points per patient for the measured variables. This demonstrates that intra-individual differences over time are relatively small, compared to inter-individual differences in the immunological variables that were measured.

**Figure 4:**
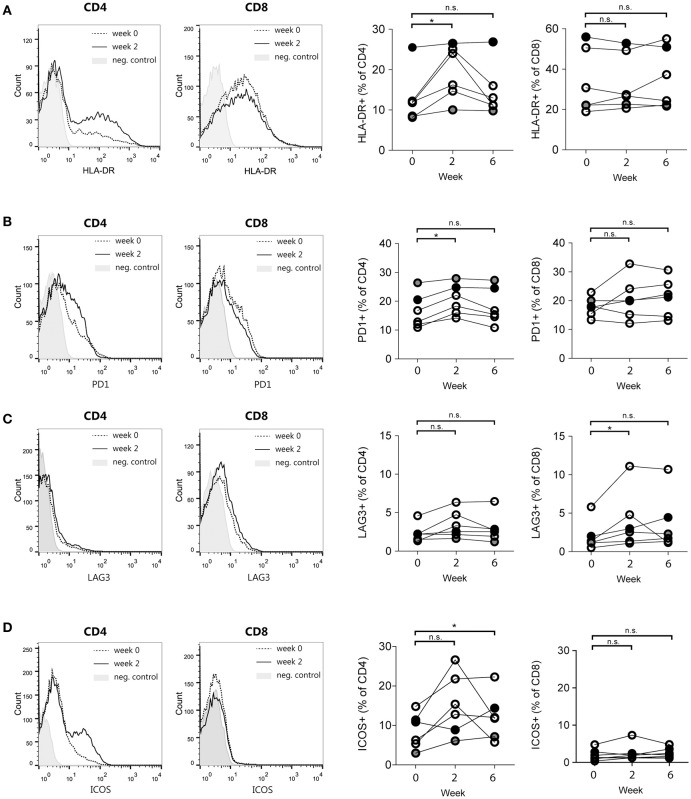
T cell activation and co-stimulatory molecule expression changes induced by DC immunotherapy. Representative histograms of flow cytometry data (left) and quantification for patients 4–9 at week 0, week 2, and week 6 after start of treatment (right). **(A)** Proportions of HLA-DR-positive CD4 and CD8 T cells. **(B)** Proportions of PD-1-positive CD4 and CD8 T cells. **(C)** Proportions of LAG3-positive CD4 and CD8 T cells and **(D)** Proportions of ICOS-positive CD4 and CD8 T cells. ^*^p < 0.05, Wilcoxon paired signed-rank test, n.s., not significant.

To further explore immunological parameters that were modulated by DC immunotherapy, we assessed the expression of the various surface markers at multiple time points. Figures 4A–D display the expression of T cell activation, co-inhibitory and co-stimulatory markers that were significantly altered either early after the start of treatment (2 weeks after the first vaccination) or later during treatment (2 weeks after the third vaccination). In particular, CD4^+^ T cells in peripheral blood showed a significant gain of HLA-DR^+^ and PD-1^+^ T cells (Figures 4A,B) after the first vaccination. After three vaccinations, we detected a significant increase of CD278 (inducible T-cell co-stimulator; ICOS) positive CD4^+^ T cells compared to baseline (Figure 4D). CD8 T cells did not show any significant increase of these markers, although CD8^+^ T cells demonstrated a significant enrichment of CD223^+^ (Lymphocyte activation gene-3; LAG-3) T cells (Figure 4C). No significant changes were observed in the following markers on both CD4^+^ and CD8^+^ T cells: CD25, CD69, CD272 (BTLA), CD137 (4-1BB), CD154 (CD40L), CD134 (OX40); (Figure S4).

In summary, these findings show that following autologous DC therapy in mesothelioma patients the frequencies of circulating CD4 T cells expressing HLA-DR, PD-1 or ICOS, as well as CD8 T cells expressing the co-inhibitory receptor LAG3 are significantly increased.

## Discussion

Here we document on the effects of treatment of MPM patients with DC immunotherapy—autologous dendritic cells loaded with an allogeneic tumor lysate prepared from five human MPM cell lines—on blood immune composition. The clinical outcomes of this first-in-human clinical trial have been published previously, and showed feasibility of the therapy, as well as promising clinical responses (9).

We used flow cytometry to analyze peripheral blood and observed an increase in absolute numbers of B cells, and CD4^+^ and CD8^+^ T cells, suggesting induction of both a cellular and humoral immune response. Additionally, we found a significant increase of HLA-DR, PD-1, and ICOS-positive CD4^+^ T cells, and an increase of LAG3-positive CD8^+^ T cells after treatment with DC vaccination therapy.

As previously shown, a vaccine-induced delayed-type hyperreactivity skin response was observed in all patients after treatment (9). Here, we confirmed that vaccine-reactive T cells were induced in the peripheral blood in three patients (two of whom receiving the lowest dose of ten million DCs). The DC immunotherapy was designed to induce a broad immune response toward multiple tumor antigens present in the tumor cell lysates. Nevertheless, for monitoring purposes that are independent of the availability of tumor material or pulsed DCs we analyzed mesothelin-specific T cells in peripheral blood during treatment. Since mesothelin was determined to be present in the tumor lysate, and expressed on the tumor cells of the patients, it was regarded to be a relevant antigen in this setting. Interestingly, after mesothelin-derived peptide-driven *in vitro* T cell propagation, CD8^+^ T cells that bind mesothelin peptide/HLA-A2 complexes could already be detected in baseline blood samples of the majority of patients, suggesting that a mesothelin-specific immune response was already present in these patients prior to therapy. However, DC vaccination did not induce changes in frequencies of the mesothelin-specific T cells in peripheral blood. Additionally, as a measure of T cell clonality, we analyzed the TCRB CDR3 length in pre- and post-treatment blood samples, which indicated that no major repertoire shifts occurred.

Both the skin responses against the vaccine in all patients after therapy, and the confirmation of these result by *in vitro* experiments in three patients, show that a vaccine-specific T cell response is induced. Furthermore, the increase in CD4^+^ and CD8^+^ T cells at week 6, in absence of a change of detectable dominant T cell clones (by TCRB CDR3 length analyses) in peripheral blood suggest that these T cell responses are of a polyclonal nature. Indeed, it has been reported that DC immunotherapy broadens, rather than skews the diversity of the T cell repertoire. In melanoma patients, not only the number of neoantigen-specific T cells increased, but also the number of clonotypes per antigen increased, indicating a further diversification of the repertoire (17).

In contrast, in studies employing vaccination with DCs pulsed with specific peptides, dominant T cell clone expansions were observed in peripheral blood (18, 19). Increases in the frequency of tumor-specific T cells were determined by IFN-γ ELISPOT (18) or HLA-A2 MHC multimers (19). The use of specific peptides to pulse DCs in these studies likely resulted in a more easily detectable clonal expansion of T cells, as compared to our strategy using complete tumor cell lysate. In one study monitoring T cells specific for a single antigen after DC vaccination using allogeneic tumor lysate increased frequencies of MART-1-specific CD8^+^ T cells were found after treatment in 3 out of 21 patients. In line with our findings, detectable numbers of these T cells were already present at baseline in 2 out of these 3 patients (20). Furthermore, as the T cells activation and the co-culture experiment showed the highest response 2 weeks at the start of treatment, we might have missed detectable changes in peripheral blood that took place before week 6. In conclusion, monitoring TCR-specificity against a single tumor-antigen (mesothelin) may support the presence of tumor-specific responses, but does not provide information on the repertoire of the tumor-specific T cell response.

Whereas a vaccine-specific response was reported to be induced in all nine patients in our clinical study (9), it remains unknown whether this response was also reactive against the autologous tumor. In previous studies with DC vaccination in mesothelioma using autologous tumor material, we found significantly increased cytotoxicity against the tumor after treatment, showing induction of a tumor-specific response by DC immunotherapy (7). In the current study, no tumor material was collected for ethical reasons, but we anticipate a comparable induction of a tumor-specific immune response based on the clinical outcomes in our patient cohort. Two partial responses were reported after DC immunotherapy, of which one remarkable response of 70% tumor reduction in a treatment-naïve patient within 6 weeks which lasted for 2 years (9).

As shown here and by others, monitoring of specific T cells in peripheral blood upon immunotherapy with a broad antigen repertoire, is challenging due to the unknown antigen composition and low frequency of individual T cell clones, and requires either very immunogenic antigens like NY-ESO-1 or MART-1 (21, 22) or *in vitro* enrichment steps, which makes it less attractive for future use in clinical practice. The current study did not address whether changes occurred in T cell reactivity in the local compartment. However, removal of tumor can be harmful in MPM due to a substantial risk of local tumor outgrowth at the intervention site (23), precluding the analysis of local tumor material for monitoring purposes. Next to T cell specificity, we investigated T cell phenotype, using extended multiplex flow cytometry. We demonstrated that treatment-related differences were most notable for CD4^+^ T cells, with an increase in surface expression of ICOS, PD-1 and HLA-DR after treatment. This signifies T cell activation, because T cells upregulate HLA-DR (24), ICOS (25) and PD-1 (26) after (TCR) stimulation.

Interestingly, both HLA-DR^+^ CD4 T cells and ICOS^+^ CD4 T cells have been described to increase after treatment with ipilimumab (anti-CTLA-4 monoclonal antibody) (27). Moreover, an increase of ICOS^+^ CD4 T cells is suggested as a possible pharmacodynamic biomarker for response to this checkpoint inhibitor (28–30). Notably, the best responding patient (#5) in our study showed the highest numbers of HLA-DR^+^ CD4^+^ and ICOS^+^ CD4^+^ T cells. Our flow cytometry panels did not allow analysis of co-expression of ICOS and HLA-DR. Others have demonstrated that ICOS^+^ T cells also expressed CD45RO (25), indicating that T cells bearing high levels of ICOS may be in an advanced maturation phase. This would be in concordance with the low proportions of CCR7 and CD27-positive T cells in our patient (#5), which are markers of less maturated T cells. In the current study patient numbers are too low to correlate the immune monitoring data with clinical outcome. Yet, our findings suggest that the ICOS^+^ CD4^+^ T cell subset may have value in the immune monitoring of future trials with DC immunotherapy. Furthermore, Fan and colleagues have described a functional role for ICOS in the anti-tumor immune response (31), providing a rationale for the combination of DC immunotherapy and engagement of ICOS-signaling in future treatments.

In conclusion, vaccination with a broad spectrum of antigens (as is the case with the allogeneic lysate used in the current study) in MPM patients, induced an increase in T cell and B cell numbers in peripheral blood. No evidence was found for a mono- or oligo-clonal T cell expansion, thus suggesting broad activation of the T lymphocytes after therapy. Additionally, changes in the frequencies of defined immune cell markers, in particular the increase of T cell activation markers in CD4^+^ T cells, demonstrated treatment-associated changes that we would propose as parameters to be included in the monitoring of DC vaccination treatments. Future studies with larger patient groups should evaluate their relation with treatment efficacy.

## Ethics statement

This study was carried out in accordance with the recommendations of the Helsinki Declaration and the Medical Research Involving Human Subjects Act, Central Committee on Research involving Human Subjects. The protocol was approved by the Central Committee on Research involving Human Subjects (NL4433000014). All subjects gave written informed consent in accordance with the Declaration of Helsinki.

## Author contributions

PdG and YK designed and performed experiments, analyzed and interpreted data and wrote the manuscript. MK-L performed and analyzed experiments. HV designed, performed and analyzed experiments. AL contributed to the design and interpretation of experiments. HV, AK, CL, JA, RD, RH contributed to design of the study and interpretation of data. All authors provided input and revised the manuscript, and all authors approved of the final version of the manuscript.

### Conflict of interest statement

JA reports receiving commercial research grants from Amphera, holds ownership interest (including patents) in Ampera BV, and is a consultant/advisory board member for Amphera. The remaining authors declare that the research was conducted in the absence of any commercial or financial relationships that could be construed as a potential conflict of interest.
