# Supplementary material for: Autologous Dendritic Cell Therapy in Mesothelioma Patients Enhances Frequencies of Peripheral CD4 T Cells Expressing HLA-DR, PD-1, or ICOS
Source: Front Immunol. 2018 Sep 7;9:2034. doi: 10.3389/fimmu.2018.02034 (PMC6137618; doi:10.3389/fimmu.2018.02034)
Supplement: Supplementary file 1 [file Table_1.DOCX]

*Supplementary Material*

**Autologous dendritic cell therapy in mesothelioma patients enhances frequencies of peripheral CD4 T cells expressing HLA-DR, PD-1 or ICOS**

*De Goeje PL#, Klaver Y#, Kaijen-Lambers MEH, Langerak AW, Vroman H, Kunert A, Lamers CH, Aerts JGJV, Debets R*, Hendriks RW**

# Contributed equally

* Correspondence: [j.debets@erasmusmc.nl](mailto:j.debets@erasmusmc.nl) and [r.hendriks@erasmusmc.nl](mailto:r.hendriks@erasmusmc.nl)

**Table S1: Patient characteristics***

|  |  |  |  |  |  |  | Skin responses to DCK, DC and lys † | | | Presence Msln-specific T cells in skin biopsy ‡ | |
| --- | --- | --- | --- | --- | --- | --- | --- | --- | --- | --- | --- |
| Patient | Pretreatment (chemo) | DC dose (per vaccination) | Number of vaccinations | Best response (RECIST) | HLA haplotype | Msln expression diagnostic biopsy^#^ | DCK | DC | lys | Msln peptide A | Msln peptide B |
| 1 | No | 10 million | 5 | SD | A1/A2 | ++ | + | - | - | - | - |
| 2 | No | 10 million | 5 | SD | A2/A68 | ++ | + | - | + | + | + |
| 3 | Yes | 10 million | 5 | SD | A2/A2 | ++ | + | - | - | - | - |
| 4 | Yes | 25 million | 5 | PR | A1/A2 | ++ | + | - | - | *ND* | *ND* |
| 5 | No | 25 million | 5 | PR | A3/A68 | ++ | + | + | - | *ND* | *ND* |
| 6 | Yes | 25 million | 5 | SD | A1/A2 | ++ | + | + | - | - | - |
| 7 | No | 50 million | 4 | SD | A2/A24 | *ND* | + | + | - | + | + |
| 8 | Yes | 50 million | 5 | SD | A2/A24 | ++ | + | + | - | + | + |
| 9 | Yes | 50 million | 4 | SD | A2/A32 | + | + | + | - | + | + |

* Patient characteristics are further detailed Aerts et al (2017)
Abbreviations: DCK, DC’s pulsed with lysate and keyhole limpet hemocyanin (KLH); DC, DC’s pulsed with lysate only; lys, tumor lysate; Msln, mesothelin; ND, not determined; SD, stable disease; PR, partial response.
# ++ = >75% positive tumor cells; + = 25-75% positive tumor cells;
† positive (+) if induration > 2 mm 48 hours after injection.
‡ positive (+) when dextramer-binding CD8 T cell frequency ≥2x negative controls

**Table S2:** List of antibodies used for multiplex flow cytometry

| **Marker** | **Label** | **Supplier** | **Cat.no** | **Clone** | **Panel*** |
| --- | --- | --- | --- | --- | --- |
| **CD3** | FITC | BD Biosciences | 345764 | SK7 | 1 |
| **CD3** | Pacific Blue | BD Biosciences | 558117 | UCHT1 | 2,3,4,5 |
| **CD4** | PE-Cy7 | BD Biosciences | 348809 | SK3 | 1 |
| **CD4** | BV510 | BD Biosciences | 560769 | RPA-T4 | 2,3,4,5 |
| **CD8** | APC-Cy7 | BD Biosciences | 348813 | SK1 | 1 |
| **CD8** | PerCP | BD Biosciences | 345774 | SK1 | 2,3,4,5 |
| **CD19** | APC | eBioscience | 17-0199-42 | HIB19 | 1 |
| **CD25** | APC | BD Biosciences | 340907 | 2A3 | 3 |
| **CD27** | APC-Cy7 | BD Biosciences | 560222 | M-T271 | 2 |
| **CD28** | APC | BD Biosciences | 559770 | CD28.2 | 2 |
| **CD45** | PerCP | BD Biosciences | 345809 | 2D1 | 1 |
| **CD45RA** | PE-Cy7 | BD Biosciences | 337186 | L48 | 2 |
| **CD56** | PE | Dako | R7251 | C5,9 | 1 |
| **CD57** | FITC | BD Biosciences | 333169 | HNK-1 | 2 |
| **CD69** | PE-Cy7 | BD Biosciences | 335792 | L78 | 3 |
| **CD127** | PE | BD Biosciences | 557938 | hIL-7R-M21 | 3 |
| **CD134 (OX40)** | FITC | eBioscience | 11-1347-42 | ACT35 (ACT-35) | 5 |
| **CD137 (4-1BB)** | PE | eBioscience | 12-1379-42 | 4B4 (4B4-1) | 5 |
| **CD152 (CTLA-4)** | APC | BD Biosciences | 555855 | BNI3 | 4 |
| **CD154 (CD40L)** | APC-Cy7 | Biolegend | 310822 | 24-31 | 5 |
| **CD223 (LAG3)** | PE-Cy7 | eBioscience | 25-2239-42 | 3DS223H | 4 |
| **CD272 (BTLA)** | PE | Biolegend | 344506 | MIH26 | 4 |
| **CD278 (ICOS)** | PE-Cy7 | eBioscience | 25-9948-42 | ISA-3 | 5 |
| **CD279 (PD-1)** | APC-Cy7 | Biolegend | 329922 | EH12.2H7 | 4 |
| **CD366 (TIM-3)** | FITC | eBioscience | 11-3109-42 | F38-2E2 | 4 |
| **CCR7** | PE | R&D | FAB197P | 150503 | 2 |
| **HLA-DR** | APC-Cy7 | BD Biosciences | 335831 | L243 | 3 |
| **TCRγδ** | FITC | BD Biosciences | 347903 | 11F2 | 1 |

*Panel 1: Absolute numbers, Panel 2: Maturation, Panel 3: Activation/Tregs, Panel 4: Co-inhibitory markers, Panel 5: Co-stimulatory markers


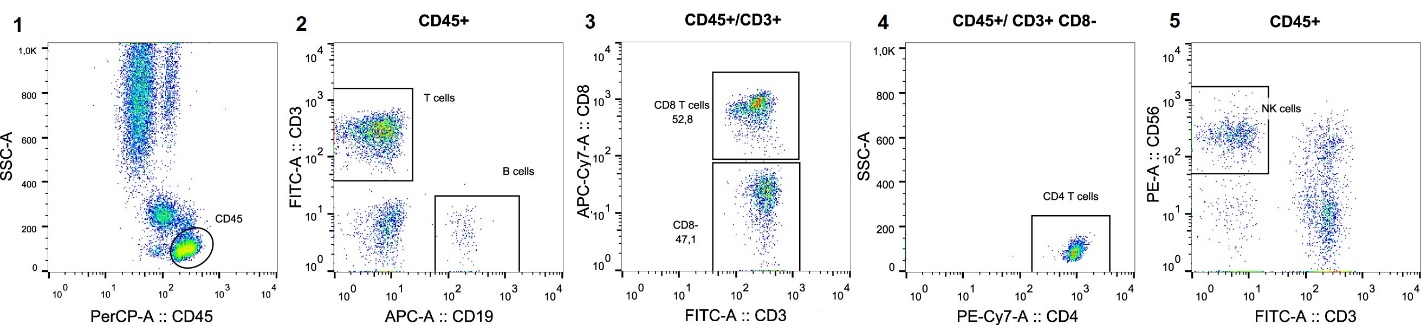


**Supplementary figure S1:** Example of gating strategy preceding the enumeration of immune cell populations. Lymphocytes were defined as CD45+, SSC-A^low^ (plot1); from CD45+, SSC-A^low^ lymphocytes, T cells (CD3+CD19-) and B cells (CD19+CD3-) were defined (plot 2). T cells were further subdivided in CD8+ T cells, and CD4+ T cells (plot3 and 4). Within the CD45+, SSC-A^low^ lymphocytes, NK cells were defined as CD56+CD3- (plot 5).


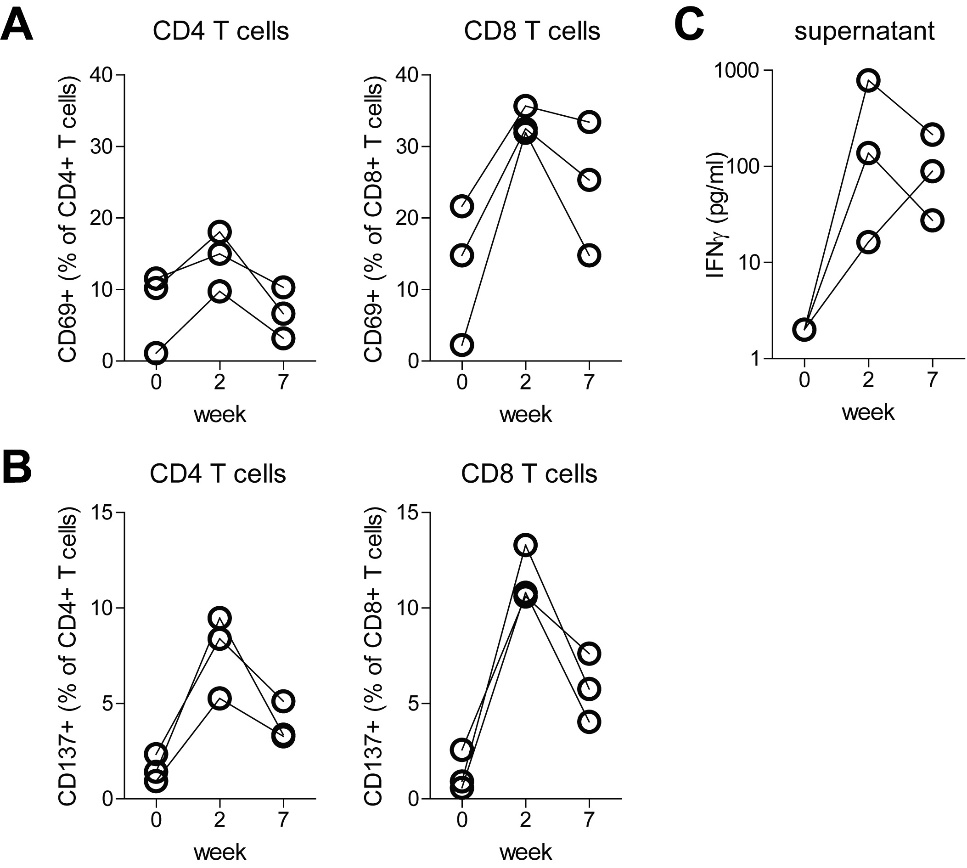


**Supplementary figure S2:** Activation of post-therapy T cells after co-culture with lysate-pulsed DCs. Lysate-pulsed DCs were co-cultured for 24h with autologous PBMC obtained at week 0, 2 or 7. A) Proportions of CD4 and CD8 T cells expressing CD69, as determined by flow cytometry. B) Proportions of CD4 and CD8 T cells expressing CD137, as determined by flow cytometry. C) Concentration of IFNγ in the culture supernatant after 24h, as determined by ELISA.


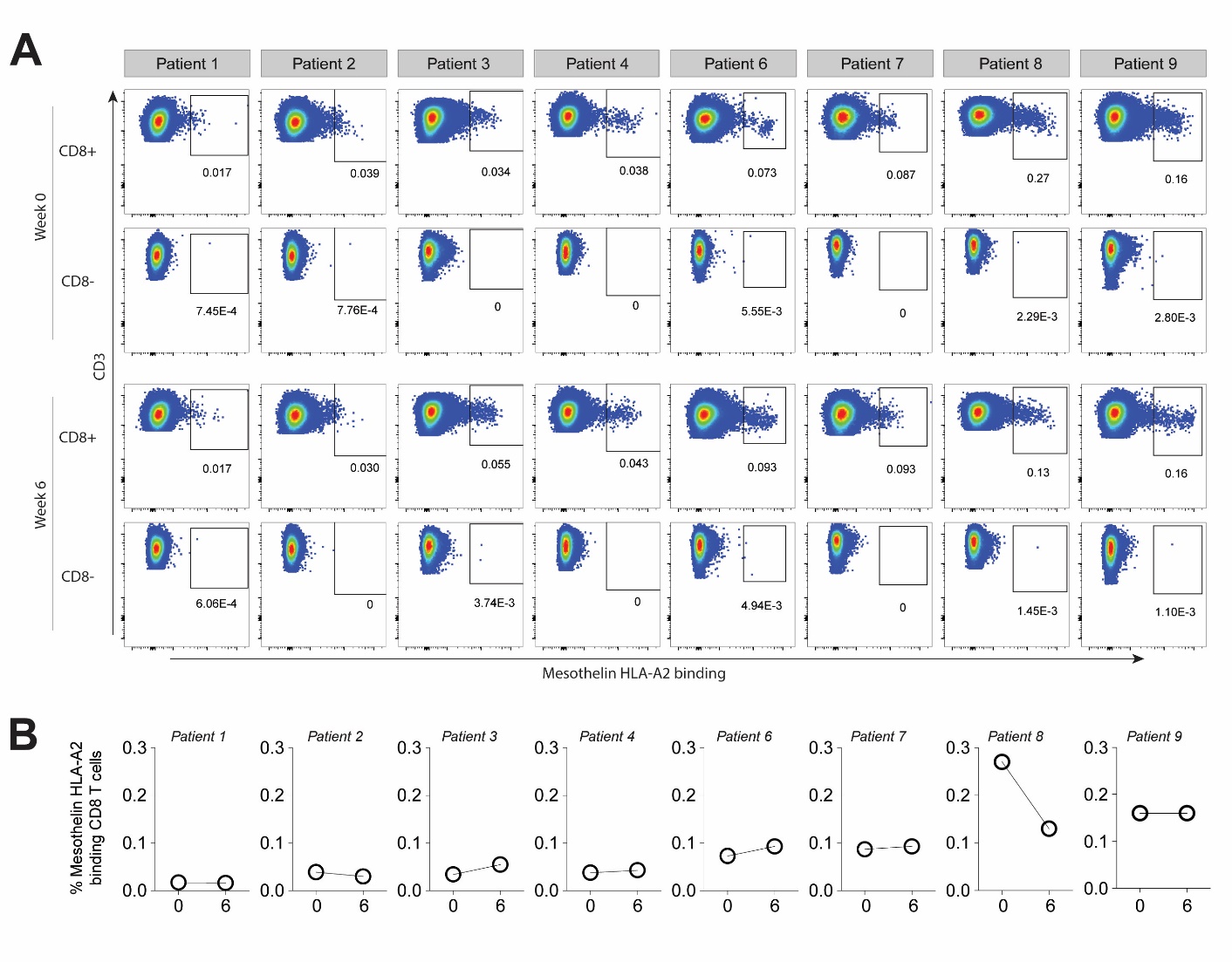


**Supplementary figure S3:** **Mesothelin peptide A-specific CD8 T cells in HLA-A2 positive patients.** A) Peripheral blood samples were collected at base line (week 0) and 2 weeks after the third vaccination (week 6). CD8 T cells were propagated in four culture cycles with mesothelin-peptide loaded aAPC after which mesothelin-peptide/HLA-A2 dextramers were used to detect mesothelin specific CD8 T cells. B) Values of gated dextramer positive CD8 T cells - as shown in panel A - presented as proportions of mesothelin-B HLA-A2-binding cells of total CD8 T cells. Gating was based on the negative controls: FMO staining and the non-CD8 T cell population. All HLA-A2 positive patients are shown; patient 5 was excluded (haplotype HLA-A3/HLA-A68).


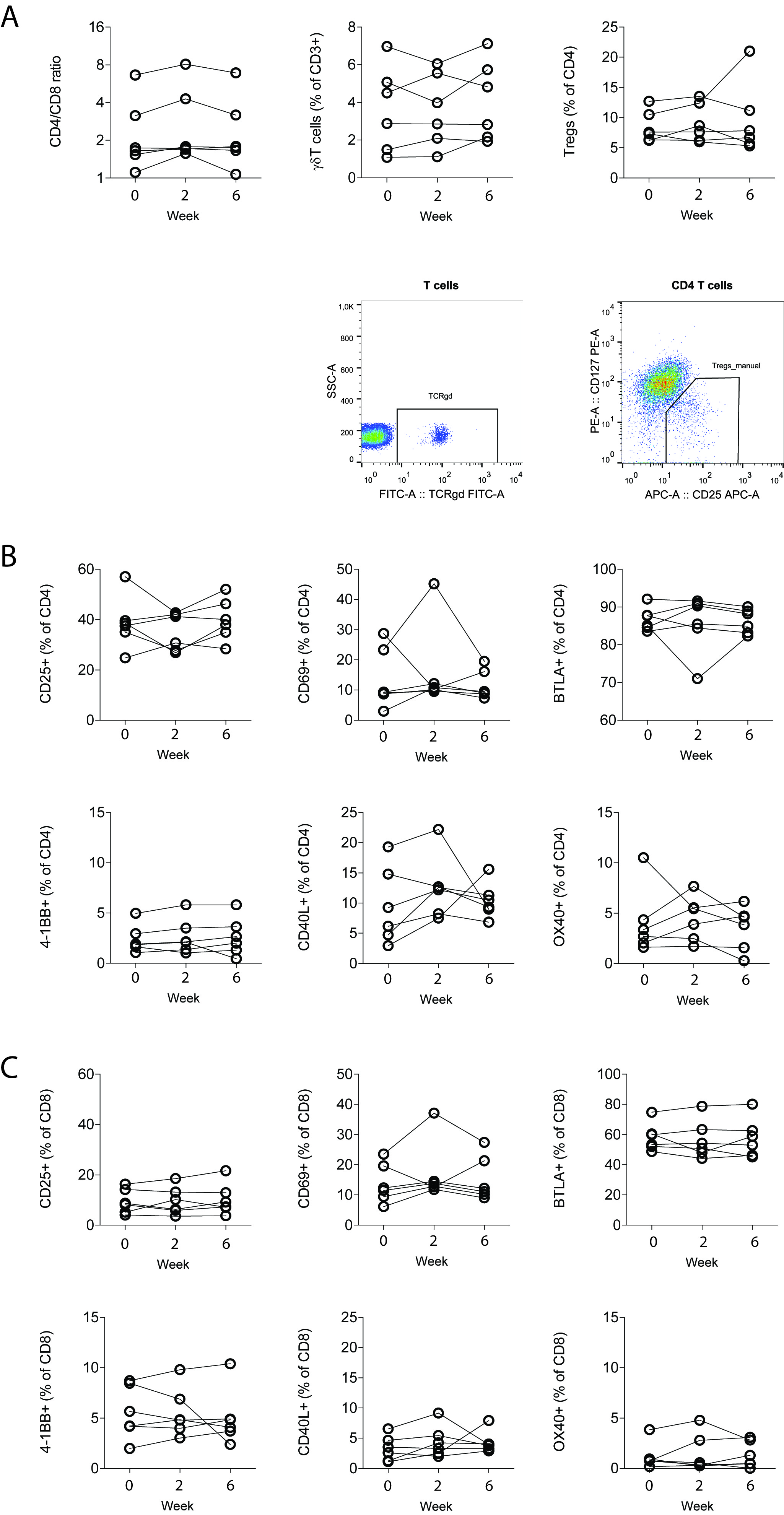


**Supplementary figure S4:** Additional frequencies of T cell populations or ratios, as well as expression of cell surface markers, showing no change between week 0, 2 and 6. A) CD4/CD8 T cell ratio, γδT cells and regulatory T cells (Treg) percentages (upper panels) and representative flow cytometry plots (lower panels; γδT cell and Treg). B) CD4 T cells positive for CD25, CD69, CD272 (BTLA), CD137 (4-1BB), CD154 (CD40L) and CD134 (OX40). C) CD8 T cells positive for CD25, CD69, CD272 (BTLA), CD137 (4-1BB), CD154 (CD40L) and CD134 (OX40)
